# Radical cation scavenging activity of berberine bridge enzyme-like oligosaccharide oxidases acting on short cell wall fragments

**DOI:** 10.1038/s41598-023-31335-y

**Published:** 2023-03-13

**Authors:** Anna Scortica, Valentina Scafati, Moira Giovannoni, Manuel Benedetti, Benedetta Mattei

**Affiliations:** Department of Life, Health and Environmental Sciences, University of L’Aquila, 67100 L’Aquila, Italy

**Keywords:** Biotic, Polysaccharides, Biocatalysis

## Abstract

Oligogalacturonide-oxidases (OGOXs) and cellodextrin-oxidase (CELLOX) are plant berberine bridge enzyme-like oligosaccharide-oxidases (OSOXs) that oxidize, respectively, oligogalacturonides (OGs) and cellodextrins (CDs), thereby inactivating their elicitor nature and concomitantly releasing H_2_O_2_. Little is known about the physiological role of OSOX activity. By using an ABTS^·+^-reduction assay, we identified a novel reaction mechanism through which the activity of OSOXs on cell wall oligosaccharides scavenged the radical cation ABTS^·+^ with an efficiency dependent on the type and length of the oxidized oligosaccharide. In contrast to the oxidation of longer oligomers such as OGs (degree of polymerization from 10 to 15), the activity of OSOXs on short galacturonan- and cellulose-oligomers (degree of polymerization ≤ 4) successfully counteracted the radical cation-generating activity of a fungal laccase, suggesting that OSOXs can generate radical cation scavenging activity in the apoplast with a power proportional to the extent of degradation of the plant cell wall, with possible implications for redox homeostasis and defense against oxidative stress.

## Introduction

Plants are constantly menaced by a wide array of pathogens such as viruses, bacteria and fungi. Against them, plants evolved a robust barrier composed of polysaccharides, phenols and proteins, i.e. the cell wall, and a sophisticated defense system that can be promptly activated at the occurrence. Phytopathogenic fungi decompose the cell wall by the action of several cell wall degrading enzymes (CWDEs), a large group of enzymes that includes glycoside hydrolases (GHs), lyases and oxidoreductases^1,2^. The enzymatic hydrolysis of cell wall polysaccharides results in the initial accumulation of cell wall fragments that can be perceived by plants as danger signals^3^; as the pathogen attack continues, these cell wall fragments are progressively converted into dimers and monomers that microbes use as carbon source to sustain their infection process^1,4^.

**Figure 1:**
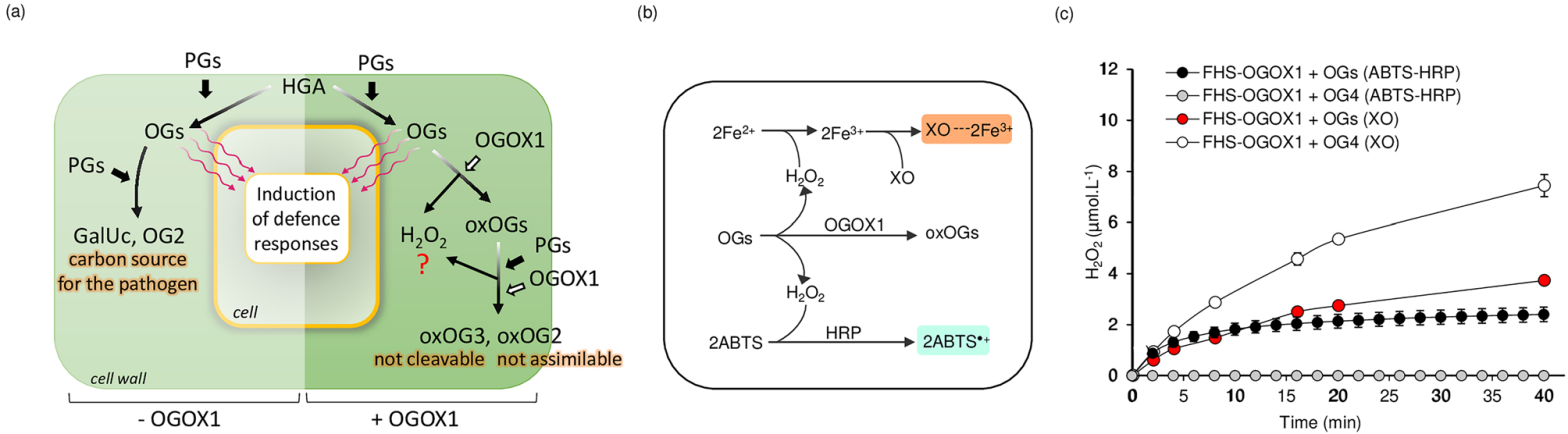
OGOX1 oxidizes galacturonan oligomers and concomitantly releases H_2_O_2_. (**a**) Potential roles of OGOX1 in plant defense: without OGOX1, PGs degrade HGA into OGs and then into OG2 and GalUc, useful carbon source for the fungus. In the presence of OGOX1, the reducing ends generated by PG activity are oxidized by OGOX1 and H_2_O_2_ is produced. The degradation products resulting from the concomitant activity of PG and OGOX1 are oxOG3 (not further cleavable by endoPGs) and oxOG2 (not assimilable by the fungal pathogen *Botrytis cinerea*). The scheme is based on the results reported in Benedetti et al.^6^. (**b**) Simplified scheme showing the reactions of (up) the xylenol orange (XO) and (down) ABTS-HRP coupled assays used for the kinetic measurements of OGOX1 using OGs as substrate. (**c**) Production of H_2_O_2_ (µmoles.L^−1^) over time at pH 5.0 by the activity of FHS-OGOX1 (4 nM) using equimolar amounts (15 µM) of OGs and OG4 by xylenol orange (XO) and ABTS-HRP coupled assay (ABTS-HRP). For the ABTS-HRP assay, the reaction mixture also included ABTS (100 µM) and HRP (0.05 g.L^−1^). Values are mean ± SD (N = 3). [*ABTS* 2,2'-azino-bis (3-ethylbenzothiazoline-6-sulfonic acid), *GalUc* D-galacturonic acid, *FHS-OGOX1* flag-his-sumoylated oligogalacturonide-oxidase 1 from *A. thaliana*, *HGA* homogalacturonan, *HRP* horseradish peroxidase, *OGs* oligogalacturonides, *OG2* di-galacturonic acid, *OG4* tetra-galacturonic acid, *OGOX1* oligogalacturonide-oxidase 1 from *A. thaliana*, *PG* polygalacturonase, *XO* xylenol orange, *oxOGs* oxidized OGs, *oxOG2* oxidized di-galacturonic acid, *oxOG3* oxidized tri-galacturonic acid].

The identification of different berberine bridge enzyme-like (BBE-l) proteins from *Arabidopsis thaliana* as specific oligosaccharide oxidases (OSOXs) paved the way to novel insights in plant immunity. To date, BBE-l proteins acting as true OSOXs are oligogalacturonide-oxidases 1–4 (OGOX1-4) and cellodextrin-oxidase (CELLOX). The four OGOX isoforms and CELLOX are capable of oxidizing, respectively, oligogalacturonides (OGs) and cellodextrins (CDs), thereby inactivating their elicitor nature and concomitantly releasing H_2_O_2_, a molecule with multiple physiological roles^5^. Besides an increased recalcitrance to the enzymatic hydrolysis displayed by the OGOX-oxidized oligosaccharides^6^, nothing is known about their involvement in other physiological processes (Fig. 1a). Protein alignment of the four OGOXs, CELLOX and other plant BBE-l proteins with carbohydrate- and monolignol-oxidizing activities allowed the identification of amino acids critical for oxidase activity, including the residue V155/157 of OGOX1/CELLOX^6,7^ as the gatekeeper residue of the oxygen binding pocket [P(T/S)VGVGG]^8–10^. OGOX1 and CELLOX are indeed efficient in catalyzing the conversion of oligosaccharides to H_2_O_2_, with H_2_O_2_ conversion efficiencies ranging from 85 to 95%^11^. Recently, different peroxidase (POD)-coupled assays were successfully used to measure the activity of fungal oligosaccharide oxidases^12^ and Arabidopsis OGOX1^13^. In the latter study, the combined use of OGOX1 and a horseradish POD (HRP) allowed the measurement of the OG-oxidizing activity in continuous mode. By using a 2,2'-azino-bis (3-ethylbenzothiazoline-6-sulfonic acid) (ABTS)-HRP coupled assay, we demonstrated that the reactivity between OGOX1 and OGs is high with a catalytic efficiency (k*cat*/KM) ranging from 3.4 × 10^5^ M^−1^.sec^−1^ (pH 5.0) to 7.9 × 10^5^ M^−1^.sec^−1^ (pH 8.5)^13^, suggesting a rapid regeneration of the oxidized flavin cofactor typical of oxidases^14,15^. Subsequently, the capability of OSOXs of converting the hydrolysing activity of microbial GHs into a controlled H_2_O_2_-dependent oxidative signal was demonstrated in vitro, by setting up an enzymatic machinery composed of a microbial GH, a plant OSOX and a plant POD^11^. The main requirement for the functioning of such machinery was that the GH/OSOX pair shared the same substrate specificity, i.e. capability of hydrolysing/oxidizing the same cell wall carbohydrate.

Investigating the physiological role of OSOXs in vivo is challenging. The complexity of the catalysed reactions and the redundancy of several BBE-l members within the same plant species make the classical biochemical and genetic approaches not conclusive^6,15^. In this context, enzymatic studies can reveal additional features of OSOXs, thereby suggesting potential physiological roles that can be investigated by targeted in vivo experiments.

By using an ABTS^·+^-reduction assay, we identified here a novel reaction mechanism through which the activity of OSOXs on cell wall oligosaccharides scavenged the radical cation ABTS^·+^ with an efficiency dependent on the type and length of the oxidized oligosaccharides. In contrast to the oxidation of longer cell wall oligosaccharides (degree of polymerization from 10 to 15), the activity of OSOXs on short cell wall oligomers (degree of polymerization ≤ 4) successfully counteracted the radical cation-generating activity of a fungal laccase, suggesting that OSOXs can generate radical cation scavenging activity in the apoplast with a power proportional to the extent of plant cell wall degradation.

## Results

### Heterologous expression of FHS-OSOXs in *Pichia pastoris*

The flag-his-sumoylated form of CELLOX (FHS-CELLOX) was obtained as previously described^11^. The sequence encoding the flag-his-sumoylated form of OGOX1, here referred to as FHS-OGOX1, was cloned under the control of the methanol-inducible promoter AOX and was expressed in *P. pastoris*. Purification was performed in a single step by immobilized metal ion affinity chromatography (IMAC) and the fractions containing the eluted enzyme were pooled into one sample. The UV–visible absorption spectrum of the protein preparation revealed the typical double absorption peak of FAD cofactor (374 and 450 nm), indicating that FHS-OGOX1 is indeed a flavoenzyme, whereas the purity grade of protein preparation (6 µM) was around 90% (Fig. S1a). The comparison of different amounts of FHS-OGOX1, as previously determined by UV-absorbance (Fig. S1a), with a standard curve of BSA by an SDS-PAGE/Coomassie blue staining analysis confirmed the high purity of the enzyme (Fig. S1b). The final protein yield obtained from 2-L culture of the highest expressing transformant was 1.5–2 mg. The specific activities of FHS-OGOX1 on OGs and two different short OG-oligomers, i.e. tri-galacturonic acid (OG3) and tetra-galacturonic acid (OG4), were 1.6 ± 0.2 (vs. OGs), 0.2 ± 0.01 (vs. OG3) and 2.3 ± 0.2 µmol H_2_O_2_.min^−1^.mg^−1^ (vs. OG4) (Fig. S1c).

### The activity of OSOXs on short oligosaccharides scavenged the radical cation ABTS^·+^

**Figure 2:**
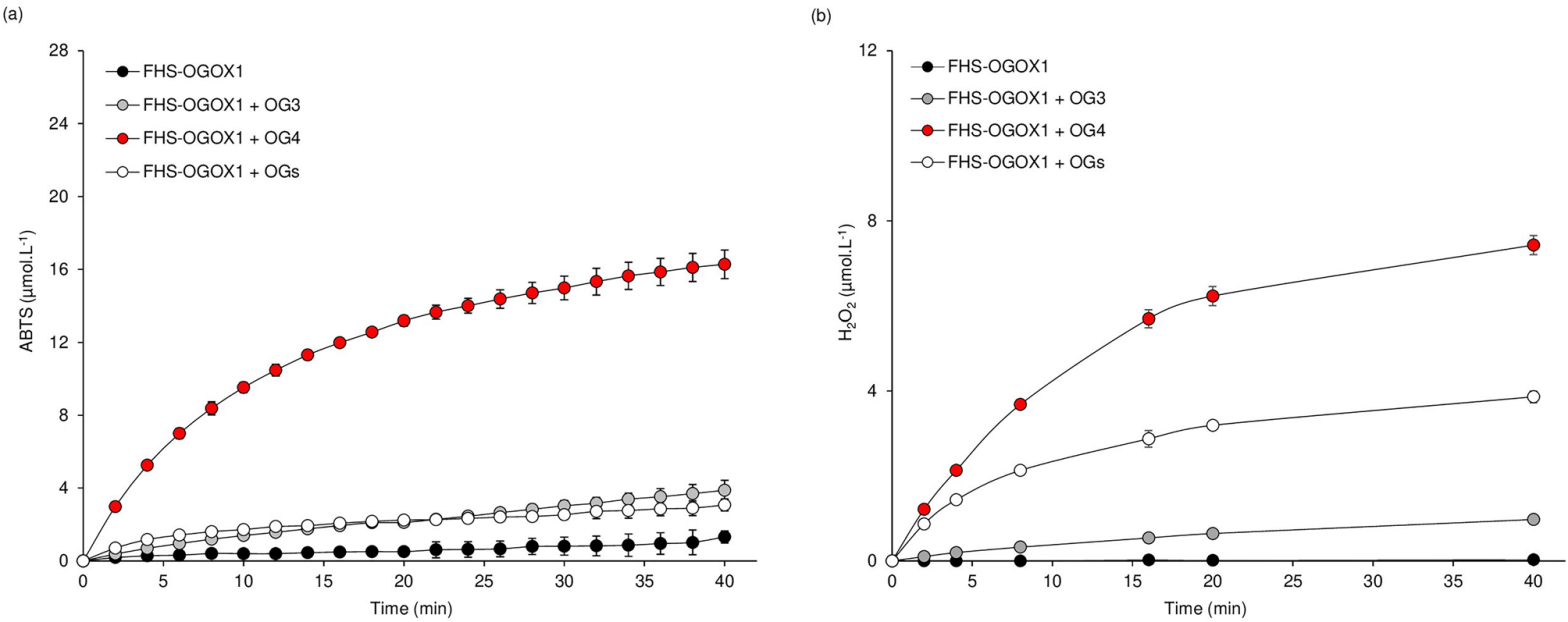
Scavenging activity of FHS-OGOX1 and short OG-oligomers towards the radical cation ABTS^·+^. (**a**) Production of ABTS (µmol.L^−1^) over time at pH 5.0 by the activity of FHS-OGOX1 (4 nM) using equimolar amounts (15 µM) of OG3, OG4 and OGs in the presence of ABTS^·+^-ABTS mixture (90–20 µM). (**b**) Production of H_2_O_2_ (µmol.L^−1^) over time at pH 5.0 by the activity of FHS-OGOX1 (4 nM) using equimolar amounts (15 uM) of OG3, OG4 and OGs. Values are mean ± SD (N = 3). [*ABTS* 2,2'-azino-bis (3-ethylbenzothiazoline-6-sulfonic acid), *FHS-OGOX1* flag-his-sumoylated oligogalacturonide-oxidase 1, *OGs* oligogalacturonides, *OG4* tetra-galacturonic acid, *OG3* tri-galacturonic acid].

**Figure 3:**
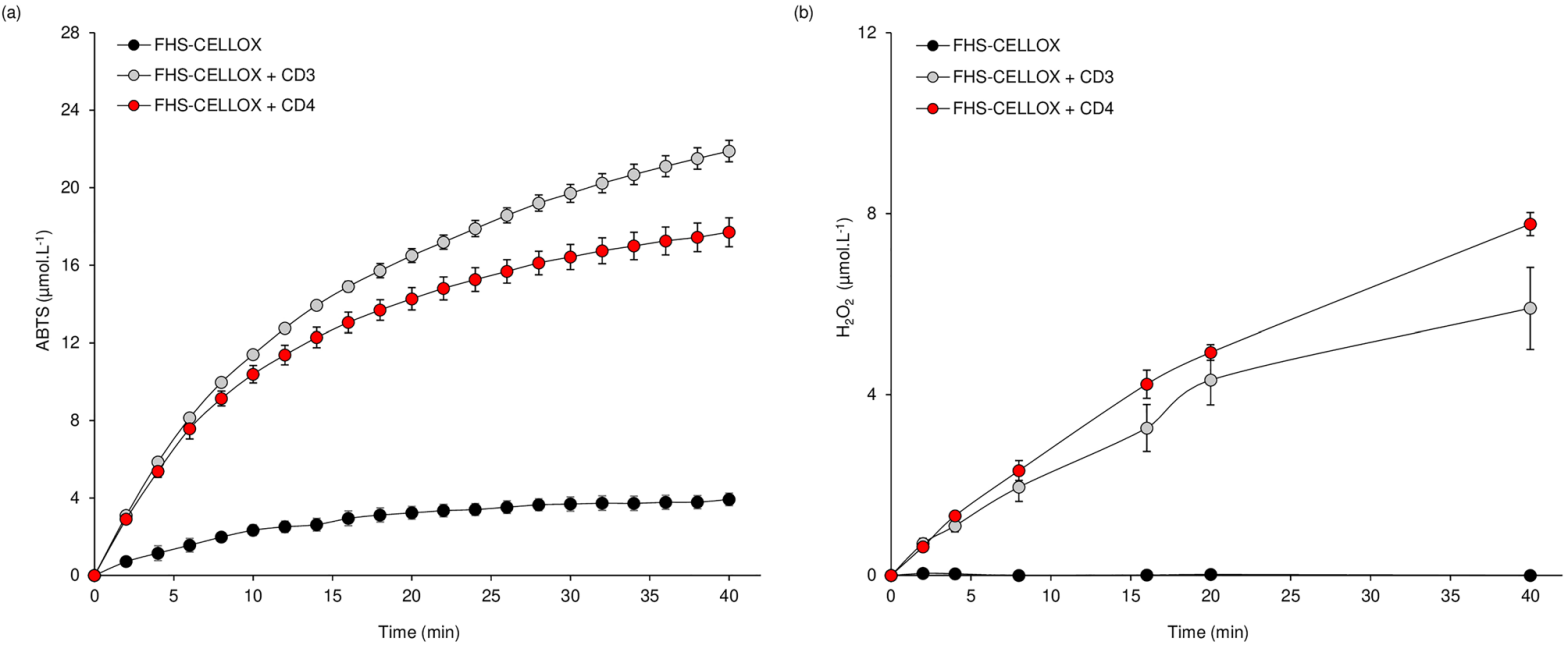
Scavenging activity of FHS-CELLOX and short CD-oligomers towards the radical cation ABTS^·+^. (**a**) Production of ABTS (µmol.L^−1^) over time at pH 5.0 by the activity of FHS-CELLOX (16 nM) using equimolar amounts (15 µM) of CD3 and CD4 in the presence of ABTS^·+^-ABTS mixture (90–20 µM). (**b**) Production of H_2_O_2_ (µmol.L^−1^) over time at pH 5.0 by the activity of FHS-CELLOX (16 nM) using equimolar amounts (15 µM) of CD3 and CD4. Values are mean ± SD (N = 3). [*ABTS* 2,2'-azino-bis (3-ethylbenzothiazoline-6-sulfonic acid), *CD4* cellotetraose, *CD3* cellotriose, *FHS-CELLOX* flag-his-sumoylated cellodextrin-oxidase].

To date, the activity of OSOXs can be evaluated by two different methods, i.e. the xylenol orange assay^6,7^ and the ABTS-HRP coupled assay^13^ (Fig. 1b). Differently from the xylenol orange assay, the ABTS-HRP coupled assay was unable to detect the activity of FHS-OGOX1 on OG4 (Fig. 1c). To deeper investigate this surprising result, we modified the buffer composition of the ABTS-HRP coupled assay by using chemically oxidized ABTS^·+^ as substrate. In brief, the starting amount of ABTS was converted into 82% ABTS^·+^ and 18% ABTS by using K_2_S_2_O_8_ as ABTS-activator and, importantly, HRP was excluded from the assay, thus reducing the number of experimental variables in the enzymatic reaction. Moreover, the concomitant presence of ABTS^·+^ and ABTS in the buffer allowed to identify all the ongoing redox reactions of the ABTS/ABTS^·+^ pair that, otherwise, could not be clearly detected in the presence of only one species (Fig. 1b). The use of such assay, hereafter referred to as ABTS^·+^-reduction assay, revealed that the activity of FHS-OGOX1 on OG4, and at lesser extent on OG3 and OGs, decreased the amount of radical cation ABTS^·+^ over reaction time (Fig. 2a). This result indicated that the lack of oxidizing activity of FHS-OGOX1 on OG4 as revealed by the ABTS-HRP coupled assay (Fig. 1c) was due to a side-reaction consisting in a time-dependent scavenging of ABTS^·+^ (Fig. 2a). In parallel, the amount of H_2_O_2_ as produced by FHS-OGOX1 was evaluated under the same reaction conditions (i.e. same pH value and enzyme/substrate concentration) by the xylenol orange assay (Fig. 2b). A time-dependent reduction of ABTS^·+^ occurred also in the presence of FHS-CELLOX and two different short CD-oligomers, i.e. cellotetraose (CD4) and cellotriose (CD3) (Fig. 3a). Also in this case, the amount of H_2_O_2_ as produced by FHS-CELLOX was evaluated under the same reaction conditions (i.e. same pH value and enzyme/substrate concentration) by the xylenol orange assay (Fig. 3b). Although OGOX1 and CELLOX are characterized by different enzymatic properties^6,7,11^, the ABTS^·+^-reduction assay was set up using concentrations of enzymes and substrates that produced H_2_O_2_ at similar rates, making it easier to compare the ABTS^·+^-scavenging activity of the two FHS-OSOXs. Our results indicated that the activities of the different FHS-OSOX/oligosaccharide combinations scavenged the radical cation ABTS^·+^ to an extent inversely related to the length of each oxidized oligosaccharide (Figs. 2, 3). For example, the scavenging activity of the FHS-OGOX1/OGs combination was comparable to that of the FHS-OGOX1/OG3 combination (Fig. 2a) although, in the absence of ABTS^·+^, the oxidizing activity of FHS-OGOX1 on OGs is by far higher than that on OG3 (Fig. 2b, Fig. S1c). Similarly, the scavenging activity of the FHS-CELLOX/CD3 combination was significantly higher than that of the FHS-CELLOX/CD4 combination (Fig. 3a) although, in the absence of ABTS^·+^, the oxidizing activity of FHS-CELLOX on CD4 is higher than that on CD3 (Fig. 3b). Notably, the type of oxidized oligosaccharides was also important since the two different FHS-CELLOX/CD-oligomer combinations produced the highest ABTS^·+^-scavenging activities (Fig. 3a).

### The radical cation scavenging activity towards ABTS^·+^ requires an ongoing activity of OSOX on short oligosaccharides

ABTS is also used as substrate to evaluate the Trolox Equivalent Antioxidant Capacity (TEAC) of several antioxidant molecules^16^. Since the reaction catalyzed by OSOXs is highly complex^3,6,13^ and different by-products could be involved, additional experiments were performed in order to better elucidate this reaction mechanism by using FHS-OGOX1/OG4 as representative FHS-OSOX/short oligomer combination (Fig. S2). The absence of a clear scavenging effect in the presence of FHS-OGOX1, H_2_O_2_, OG4, [H_2_O_2_ + OG4] or [H_2_O_2_ + oxidized OG4 (oxOG4)] demonstrated that an ongoing activity of FHS-OGOX1 on OG4 is essential for the time-dependent reduction of ABTS^·+^ (Fig. S2). Unfortunately, the ABTS^·+^-reduction assay contains an amount of ABTS^·+^ (90 µmol.L^−1^) that interferes with the xylenol orange, impeding its utilization for a direct measurement of H_2_O_2_ during the reaction of scavenging. To detect any eventual production of H_2_O_2_, three different FHS-OGOX1/oligomer combinations were assayed in the presence of an excess of HRP, here used to counter-oxidize the scavenged ABTS^·+^ by consuming the H_2_O_2_ produced with the different OSOX/oligomer combinations (Fig. 1b)^13^. In the presence of HRP, the ABTS^·+^-scavenging activity of both FHS-OGOX1/OG3 and FHS-OGOX1/OG4 combinations remained the same, whereas an opposite activity (production of ABTS^·+^) was observed for the FHS-OGOX1/OGs pair (Fig. S3). Based on this result (Fig. S3), the presence of H_2_O_2_ was detected only in the reaction containing FHS-OGOX1/OGs, i.e. the combination characterized by the lowest ratio between ABTS^·+^-scavenging activity (Fig. 2a) and H_2_O_2_-producing activity (Fig. 2b).

### The ABTS-oxidizing activity of a fungal laccase is counteracted by the activity of OSOXs on short oligosaccharides

**Figure 4:**
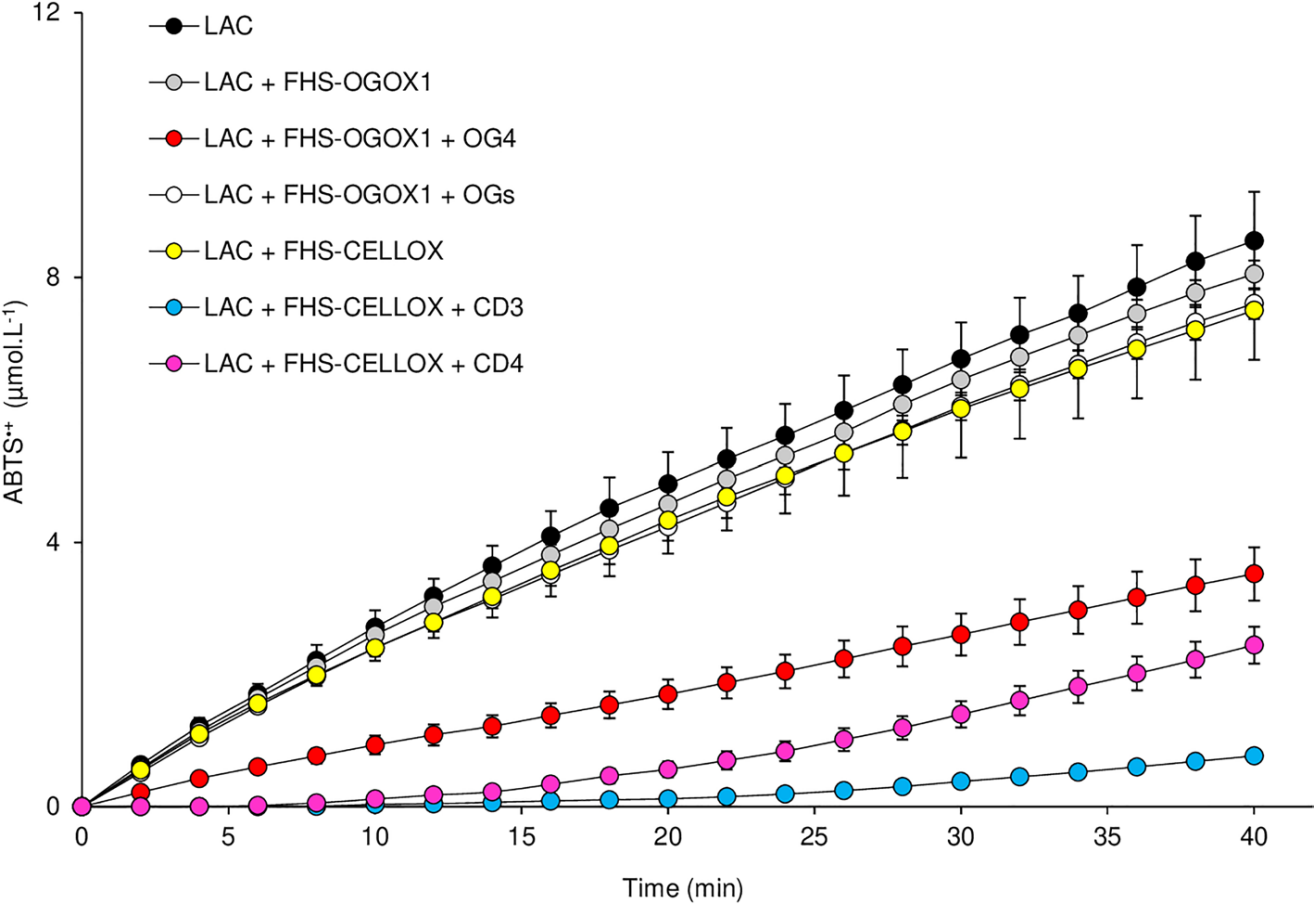
The activity of FHS-OSOXs on short oligosaccharides counteracts the laccase-catalyzed oxidation of ABTS. Production of ABTS^·+^ (µmol.L^−1^) over time at pH 5.0 by laccase from *T. versicolor* alone (5 µg.mL^−1^) and in the presence of FHS-OGOX1 (4 nM) and two different galacturonic acid oligomers (OG4 and OGs) (15 µM), and of FHS-CELLOX (16 nM) and two different cellodextrins (CD3 and CD4) (15 µM). The enzymatic reaction also included ABTS (100 µM). Laccase activity was not affected in the only presence of oligomers (not shown). Values are mean ± SD (N = 3). [*ABTS* 2,2'-azino-bis (3-ethylbenzothiazoline-6-sulfonic acid), *CD3* cellotriose, *CD4* cellotetraose, *FHS-CELLOX* flag-his-sumoylated cellodextrin-oxidase, *FHS-OGOX1* flag-his-sumoylated oligogalacturonide-oxidase 1, *LAC* laccase from *T. versicolor*, *OGs* oligogalacturonides, *OG4* tetra-galacturonic acid].

To evaluate whether the scavenging activity of OSOXs can counteract the oxidizing activity of microbial ligninases, the laccase from *Trametes versicolor* was assayed in the presence of different FHS-OSOX/oligomer combinations by using ABTS as substrate. It is worth noting that the oxidation of ABTS by laccase, differently from HRP, is not H_2_O_2_-dependent. The ABTS-oxidizing activity of laccase was strongly reduced by the FHS-CELLOX/CD3 combination and, at lesser extent, by the FHS-CELLOX/CD4 and FHS-OGOX1/OG4 combinations, whereas the FHS-OGOX1/OGs combination was ineffective (Fig. 4). Indeed, this result clearly demonstrated that the scavenging activity of OSOXs towards the laccase-catalyzed oxidation of ABTS required the presence of oxidizable short oligosaccharides. Differently from the ABTS^·+^-reduction assay, the lower amount of ABTS^·+^ (0–9 µmol.L^−1^, Fig. 4) as contained in the ABTS-oxidation assay allowed a direct measurement of H_2_O_2_ by using the xylenol orange. In the presence of laccase, the (negligible) scavenging activity of the FHS-OGOX1/OGs combination did not have any impact on the production of H_2_O_2_ whereas the strong scavenging activities of both FHS-OGOX1/OG4 and FHS-CELLOX/CD3 combinations were associated with decreased levels of H_2_O_2_ (Fig. S4). Also in this case, higher scavenging activities were related to lower levels of H_2_O_2_ and vice versa.

In conclusion, the OSOX activity on short oligosaccharides scavenged the radical cation ABTS^·+^ (Fig. S2) with an efficiency dependent on the type and length of each oxidized oligosaccharide (Figs. 2, 3, 4) and at the expense of the H_2_O_2_ as produced from their oxidation (Figs. S3, S4).

### The oxidation rate of CD3 is higher in the presence of ABTS^·+^

**Figure 5:**
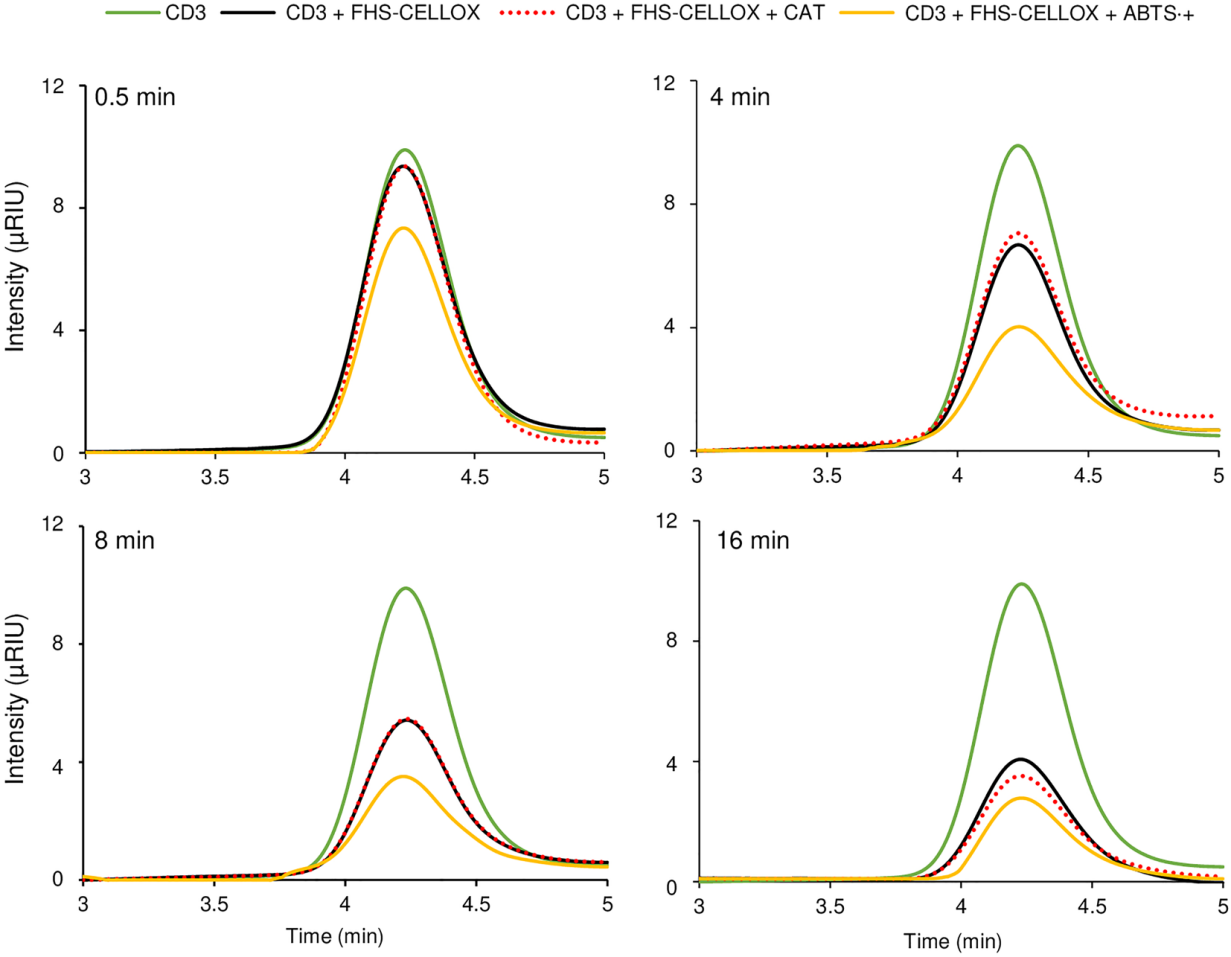
Analysis of residual CD3 upon incubation with FHS-CELLOX and ABTS^·+^. Chromatographic analysis of CD3 alone (CD3) (12 µg), CD3 incubated with FHS-CELLOX (CD3 + FHS-CELLOX), CD3 incubated with FHS-CELLOX and catalase (CD3 + FHS-CELLOX + CAT) and CD3 incubated with FHS-CELLOX and ABTS^·+^/ABTS (CD3 + FHS-CELLOX + ABTS^·+^). Analysis was performed after 0.5, 4, 8 and 16 min of reaction. CD3 and FHS-CELLOX were used at 600 µM and 0.7 µM, respectively. ABTS^·+^/ABTS pair was used at 1 mM (800/200 µM). [*ABTS* 2,2'-azino-bis (3-ethylbenzothiazoline-6-sulfonic acid), *CAT* catalase from bovine liver, *CD3* cellotriose, *FHS-CELLOX* flag-his-sumoylated cellodextrin-oxidase].

To obtain additional information on the molecular mechanism underlying the ABTS^·+^-scavenging activity of OSOXs and short oligosaccharides, the reaction with the FHS-CELLOX/CD3 combination was further analyzed. In particular, the amount of residual CD3 (i.e. non-oxidized) was evaluated after different incubation times with FHS-CELLOX by HPLC analysis, both in the presence of ABTS^·+^ and in the control reaction (i.e. without ABTS^·+^). For each reaction time tested, the amount of residual CD3 was significantly lower in the presence of ABTS^·+^ than in the control reaction, indicating that ABTS^·+^ increased the oxidation rate of CD3 by FHS-CELLOX (Fig. 5). It is worth noting that the reduced production of H_2_O_2_ in the presence of high ABTS^·+^-scavenging activity (Figs. S3, S4) could preserve OSOXs from a possible H_2_O_2_-mediated inactivation, a condition that cannot be avoided in the control reactions, i.e. where H_2_O_2_ accumulates (Figs. 2b, 3b). However, when the control reactions were performed in the presence of catalase, here used as H_2_O_2_-detoxifying enzyme, the amount of residual CD3 remained the same (Fig. 5), pointing to the radical cation ABTS^·+^ as the main responsible for the higher oxidation rate of CD3 (Fig. 5).

## Discussion

### Radical cation scavenging activity of FHS-OSOXs acting on short oligosaccharides: biochemical considerations

**Figure 6:**
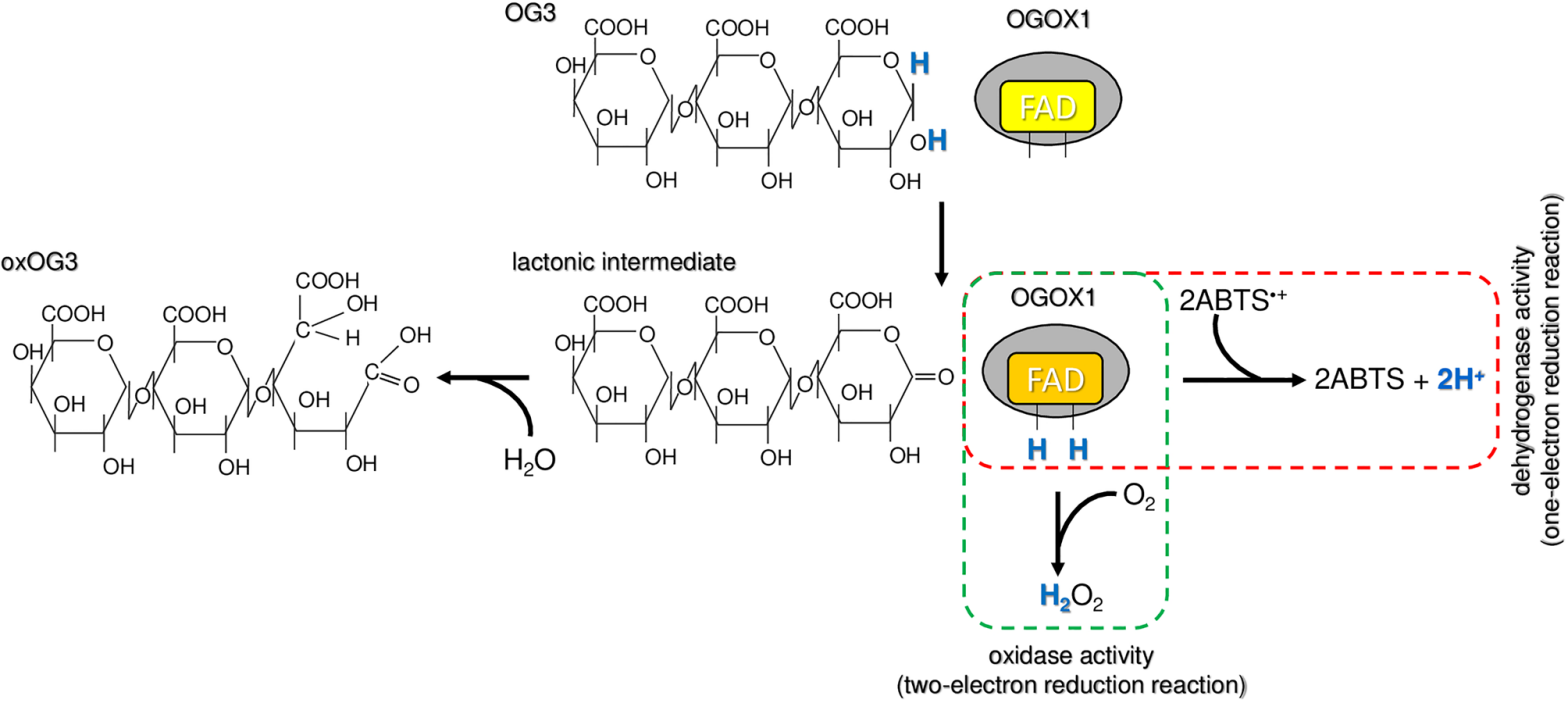
Model of the ABTS^·+^-scavenging reaction catalyzed by an OSOX/short oligosaccharide combination. In the conventional reaction (dotted green box), OGOX1 oxidizes OG3 to a lactonic intermediate by transferring two electrons to one molecule of O_2_ (oxidase activity, two-electron reduction reaction). In the ABTS^·+^-scavenging reaction (dotted red box), OGOX1 oxidizes OG3 to a lactonic intermediate by transferring two electrons to two molecules of ABTS^·+^ (dehydrogenase activity, one-electron reduction reaction). In both reactions, the lactonic intermediate is then spontaneously hydrolyzed to oxOG3 (GalUc1–4αGalUc1–4Galactaric acid). OGOX1 and OG3 are used here as representative OSOX/short oligosaccharide combination [*ABTS* 2,2'-azino-bis (3-ethylbenzothiazoline-6-sulfonic acid), *GalUc* D-galacturonic acid, *OGOX1* oligogalacturonide-oxidase 1, *OG3* tri-galacturonic acid, *oxOG3* oxidized tri-galacturonic acid].

POD-mediated oxidation of ABTS is well-documented since 1975^17^; subsequently, the use of ABTS was also extended to fungal laccases as redox mediator in lignin depolymerization^18^. To our knowledge, this is the first report describing a clear enzyme-dependent scavenging activity towards the radical cation ABTS^·+^. Although the use of ABTS as mediator in biological redox reactions^19^ can be criticized due to its synthetic nature, the scavenging activity towards ABTS^·+^ was achieved here by using nanomolar concentration of OSOXs (4–16 nM) and micromolar concentration of each oligosaccharide (15 µM); notably, the latter substrate concentration was about 150–3000 folds lower than that reported for the non-enzymatically catalyzed ABTS^·+^-reduction reactions^20^. Moreover, the radical cation scavenging activity was generated by OSOXs with different substrate specificities and was markedly dependent on the type and length of oxidized oligosaccharides (Figs. 1, 2, 3, 4). Indeed, the H_2_O_2_ produced by OSOXs on short oligosaccharides was undetectable at high ABTS^·+^ concentration (Fig. S3) or in a reduced amount at low ABTS^·+^ concentration (Fig. S4), suggesting that the reduction of ABTS^·+^ to ABTS was directly mediated by the FAD(H_2_)-cofactor of OSOXs (Fig. 6). Notably, the redox potentials of O_2_^21^ and ABTS^·+^^22^ are consistent with this hypothesis, that can explain both the lack of H_2_O_2_ production in the presence of scavenging activity (Fig. S3) and the higher oxidation rate of CD3 by FHS-CELLOX in the presence of ABTS^·+^ (Fig. 5). In the ABTS^·+^-reduction assay, the higher reactivity of reduced FAD(H_2_)-cofactor with the radical cation ABTS^·+^ may result in a more efficient FAD regeneration and then in a higher oxidation (consumption) rate of short oligosaccharides (Figs. 2a, 3a, 5). Moreover, both OSOXs displayed a slight, intrinsic radical cation scavenging activity towards ABTS^·+^ (*see* the production of ABTS in the reactions with the enzyme alone, Figs. 2a, 3a), with FHS-CELLOX showing a higher ABTS^·+^-scavenging propension than FHS-OGOX1. This effect was dependent on the type of OSOX and may also contribute to the higher ABTS^·+^ scavenging activity observed for the two FHS-CELLOX/CD combinations (Fig. 3a). In this regard, the use of direct assays capable of accurately evaluating (i) the amount of oxidized oligosaccharides and (ii) the O_2_ consumption over reaction time in the presence of the radical cation ABTS^·+^ will be fundamental, both in substrate excess and in equimolar enzyme/substrate conditions. In literature, other flavoenzymes with dual nature of oxidase and dehydrogenase have been already characterized^23,24^. As observed for the bacterial Pyranose 2-Oxidase^23^, we can conclude that OSOXs too are versatile flavoenzymes, acting as oxidases in the presence of longer oligosaccharides and O_2_ (two-electron reduction reaction), and as dehydrogenase in the presence of short oligosaccharides and radical cation ABTS^·+^ (one-electron reduction reaction) (Fig. 6). Noteworthy, the prevalence of one or the other reaction is dependent not only on the type of FAD(H_2_)-electron acceptor (e.g. ABTS^·+^) but also on its concentration with respect to the concentration of O_2_ and short oligosaccharides (Figs. S3, S4, Figs. 2a, 3a, 5).

### Radical cation scavenging activity of FHS-OSOXs acting on short oligosaccharides: physiological implications

**Figure 7:**
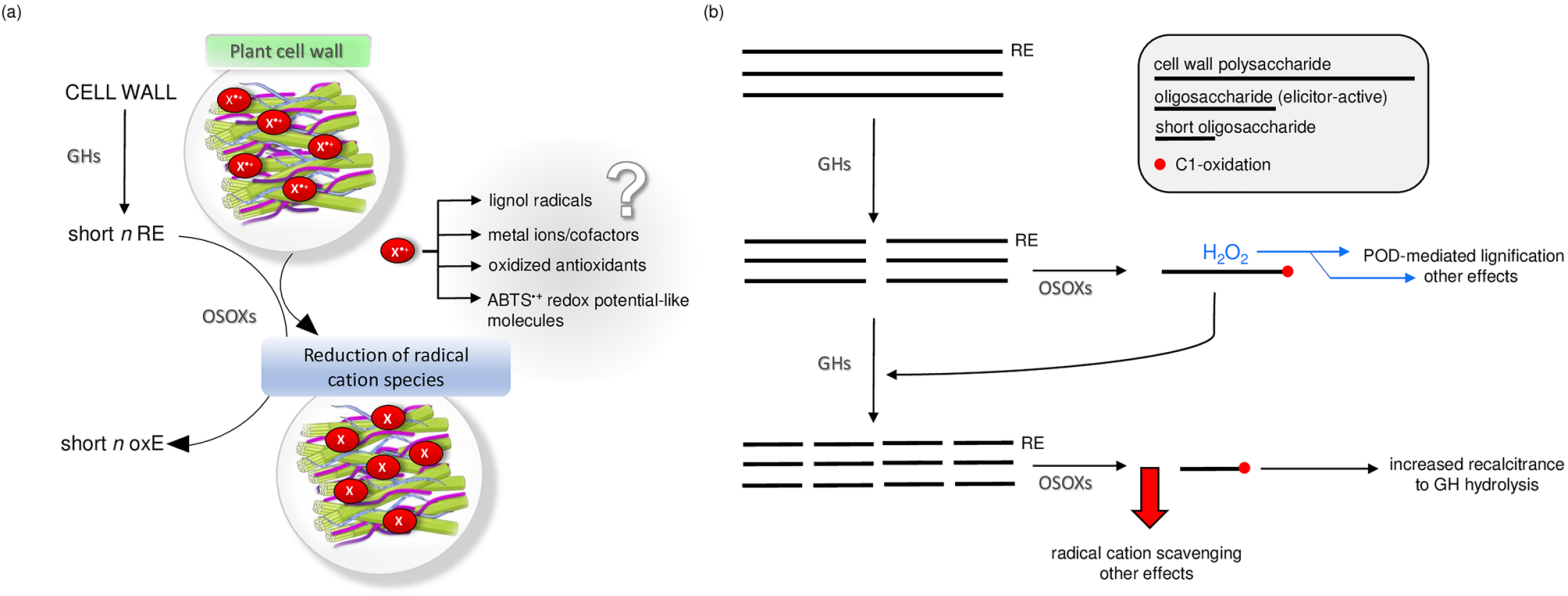
Proposed models of OSOXs as cell wall defense proteins. (**a**) Scavenging of unknown radical cations (X^·+^) by the activity of OSOXs on short cell wall oligosaccharides. (**b**) Potential contribution of reaction products from OSOX activity in the maintenance of cell wall integrity during infection. [*GH* glycoside hydrolase, *OSOX* berberine bridge enzyme-like oligosaccharide oxidase, *oxE* oxidized end, *POD* plant peroxidase, *RE* reducing end].

In vivo, the scavenging activity of different OSOX/oligomer combinations may be directed towards metal ions/cofactors, lignol radicals, oxidized antioxidants and/or unknown molecules of plant and of microbial origin with redox potentials similar/higher to that of the radical cation ABTS^·+^ (Fig. 7a).

To date, OGOX1-4 and CELLOX are the only plant BBE-l enzymes with proven oxidizing activities towards cell wall fragments with elicitor nature, i.e. OGs and CDs, and therefore they can be classified as true OSOXs. The physiological role of OSOXs is still under investigation. During the degradation of plant cell wall, the resulting cell wall fragments can be converted by OSOXs into H_2_O_2_ and oxidized oligosaccharides. By using a multiple enzyme-based assay, we have already demonstrated that H_2_O_2_ from OSOX activities may be used by extracellular PODs to reinforce the cell wall in a manner proportional to the entity of cell wall damage taken^11^. Here we found a possible involvement of OSOX activity on short cell wall oligomers as scavenger towards the radical cation-producing activity of fungal laccases, or more generally, towards the radical cation chain reactions occurring during pathogenesis. Intriguingly, cellulose and lignin are strictly associated in lignocellulose^1^, suggesting that cellulose fragments with free reducing ends could be exploited as electron source by CELLOX to contrast the radical cation-generating activity of microbial ligninases^25^ and thereby preserve the integrity of lignocellulose. The scavenging activity of the different FHS-OSOX/oligomer combinations towards ABTS^·+^ was inversely related to the length of each oxidizable oligomer, i.e. the shorter the oligomer, the higher the scavenging activity (Figs. 2, 3), suggesting that these reaction mechanisms may differently contribute to plant defense depending on the progression of the infection. For example, H_2_O_2_-dependent lignification can be mediated by extracellular plant PODs as soon as cell wall fragments are formed by microbial GHs and oxidized by plant OSOXs^11^, whereas the radical cation-scavenging activity may occur at a later infection stage, i.e. when the cell wall oligosaccharides, including those already oxidized, are further converted into smaller oligomers by microbial GHs (Fig. 7b). Indeed, a strong scavenging activity in the first phase of infection could interfere with the radical-generating activity of plant peroxidases and other H_2_O_2_-mediated reactions (Fig. S3, Fig. 7b), making understandable why the activity of FHS-OGOX1 on degradation-intermediate products (e.g., longer oligomers such as OGs) was characterized by a negligible scavenging activity.

In conclusion, our study, although limited to in vitro evidence, provides a novel perspective on how OSOXs can generate radical cation scavenging activity in the apoplast with a power proportional to the extent of degradation of plant cell wall, with implications for redox homeostasis and defense against oxidative stress during microbial infection. In the presence of short oligosaccharides, the O_2_-consuming reactions that take place in the apoplast under stress conditions may promote the scavenging activity of OSOXs towards the radical cation species of natural origin (Fig. 7a). The knowledge of this enzymatic mechanism (Fig. 6) will allow the design of in vivo experiments aimed at demonstrating the existence of the processes depicted in Fig. 7.

## Methods

### Construction of the synthetic gene encoding FHS-OGOX1 by bioinformatic tools

The gene encoding the mature OGOX1.2 isoform from *Arabidopsis thaliana* (AT4G20830.2) was fused downstream of the SUMOstar sequence developed by LifeSensor Inc. (https://lifesensors.com/) that also included the sequences encoding the FLAG- (DYKDDDDK) and 6xHis-tags (HHHHHH) (https://lifesensors.com/wp-content/uploads/2019/09/2160_2161_Pichia_SUMOstar_Manual-1.pdf). The sequence of the chimeric gene, here referred to as *FHS-OGOX1*, was codon-optimized with the codon usage of *Pichia pastoris* by using the online tool OPTIMIZER (http://genomes.urv.es/OPTIMIZER/)^26^ and was entirely synthesized by Genescript (https://www.genscript.com/) by adding the bases of the restriction sites PstI and XbaI at the 5^I^ and 3^I^ ends, respectively, of the gene. The gene was then cloned in pPICZαB expression vector (Invitrogen, San Diego, USA) in frame with the sequence encoding the yeast α factor for the secretion of recombinant proteins in the medium.

### Heterologous expression of FHS-OSOXs in *P. pastoris*

The recombinant FHS-CELLOX, i.e. a flag-his-sumoylated form of CELLOX from *A. thaliana* (AT4G20860), was expressed and purified as previously described^11^. The construct pPICZαB/FHS-OGOX1 was transformed in *E. coli* DH5α competent cells (ThermoFisher, Waltham, USA) for plasmid amplification. Then, the construct was linearized by SacI and introduced in *P. pastoris* by electroporation^27^. Multi-copy transformants were selected on solid YPDS medium [1% (w/v) yeast extract, 2% (w/v) peptone, 2% (w/v) dextrose, 1 M Sorbitol] with zeocin as antibiotic resistance marker (1 mg.mL^−1^). For protein expression, several colonies of multicopy *P. pastoris* transformants were inoculated in 5 mL of YPD medium [1% (w/v) yeast extract, 2% (w/v) peptone, 2% (w/v) dextrose] supplemented with 0.2 mg.mL^−1^ zeocin and incubated at 28 °C in a rotary shaker at 180 rpm for 72–96 h. The cultures were then centrifuged and the cell pellets were resuspended in 1.5 mL of Buffered Minimal Medium [0.1 M K-phosphate (pH 6.0), 1.34% (w/v) YNB, 4 × 10^–5^% (w/v) biotin and 0.5% (v/v) methanol] to induce the expression of FHS-OGOX1 and let them grow for additional 48 h. To detect the expression of recombinant FHS-OGOX1, different culture filtrates were evaluated by SDS-PAGE/Coomassie blue staining. For FHS-OGOX1 purification, the methanol-induced culture filtrate from the highest expressing transformant was subjected to buffer exchange (50 mM Tris–HCl pH 7.5, 500 mM NaCl, 1 mM 2-mercaptoethanol and 10 mM imidazole) and then loaded onto a HisTrap HP column (ThermoFisher, Waltham, USA). The eluted fractions containing FHS-OGOX1 were pooled and dialyzed in 50 mM Tris–HCl pH 7.5, 100 mM NaCl and 100 mM (NH_4_)_2_SO_4_. Before its use in the assays, the dialyzed FHS-OGOX1 preparation was quantified by UV–visible absorbance and then analyzed by SDS-PAGE/Coomassie blue staining. For the UV–visible spectrum, pure FHS-OGOX1 was analyzed by NanoDrop One (Thermo Fisher, Waltham, USA) before and after concentration (~ 3X) using a Vivaspin 500 centrifugal concentrator (30.000 MWCO PES) (Sartorius, Gottinga, Germany). The purity grade of the protein preparation was calculated as [(moles FAD.moles FHS-OGOX1^–1^) × 100%] (FAD, ε_450nm_ = 11,300 M^−1^.cm^−1^; FHS-OGOX1, ε_280nm_ = 78,270 M^−1^.cm^−1^). The activity of FHS-OGOX1 was evaluated by using the xylenol orange assay^6^. The activity of FHS-OGOX1 was assayed in 50 mM Na-Acetate pH 5.0 and 50 mM NaCl by using OGs, OG3 or OG4 (15 µM) as substrates in the presence of the purified enzyme (4 nM), and then expressed as µmoles of H_2_O_2_ generated per minute per mg of FHS-OGOX1. The OG mixture (degree of polymerization: 10–15; average value used for mass-to-mole conversion: 2306 g.mol^−1^) and OG3 were purchased from Biosynth Carbosynth (https://www.carbosynth.com/) whereas OG4 was purchased from ELICYTIL (https://www.elicityl-oligotech.com/). For each galacturonan oligomer stock solution (1 mM), the amount of reducing ends was confirmed by the reducing sugar-assay^28^ using different amounts of galacturonic acid as calibration curve. All the enzymatic assays described in this work were performed at apoplastic pH value (5.0) and by using an oligosaccharide concentration (15 µM) compatible with that used to trigger the defense responses in plants^13^.

### Evaluation of FHS-OGOX1 activity by ABTS-HRP coupled and xylenol orange assays

Preliminarily, the OG-oxidizing activity of FHS-OGOX1 was measured by two different spectrophotometric methods, i.e. the ABTS-HRP coupled assay^13^ and the xylenol orange assay^6^. In both assays, the activity of FHS-OGOX1 was evaluated in 50 mM Na-Acetate pH 5.0 and 50 mM NaCl by adding OGs or OG4 (15 µM) as substrates in the presence of the purified enzyme (4 nM) without stirring. In the ABTS-HRP coupled assay, the enzymatic reaction (0.2 mL) included also ABTS (100 µM) and HRP VI-A type (0.05 g.L^−1^) (P6782, Sigma-Aldrich, St. Louis, USA) whereas the absorbance at 415 nm was measured in continuous mode, subtracted to that of control reaction (basal mixture) and then converted into µmol ABTS^·+^ (ε415nm = 36 mM^−1^.cm^−1^). Conversion of µmol ABTS^·+^ into µmol H_2_O_2_ was obtained by applying the conversion factor [1.92 molecules ABTS^·+^: 1 molecule H_2_O_2_]^29^. Differently from the ABTS-HRP coupled assay, the xylenol orange assay is an end-point method^6^. In accordance with^30^, the enzymatic reactions (0.1 mL) were incubated without stirring and then blocked by adding one volume of “xylenol orange developing solution” [1 M sorbitol, 0.001 M xylenol orange, 0.0025 M (NH_4_)_2_Fe(SO_4_)_2_, 0.25 M H_2_SO_4_], thereby resulting in a drastic pH drop and enzyme inactivation. The absorbance at 560 nm from the same enzymatic reactions was measured at seven different time-points (0, 2, 4, 8, 16, 20 and 40 min), subtracted to that of control reaction (basal mixture) and converted into µmol H_2_O_2_ by interpolation with the H_2_O_2_-calibration curve. All the analysis were performed in triplicates at 25 °C.

### Evaluation of scavenging activity of FHS-OSOXs by ABTS^·+^-reduction assay

In the ABTS^·+^-reduction assay, the starting concentration of ABTS (110 µM) was converted into 90 µM ABTS^·+^ and 20 µM ABTS by using K_2_S_2_O_8_ as ABTS-activating agent. Exhaustive K_2_S_2_O_8_-mediated oxidation of ABTS was achieved by incubating the reaction for 16 h at 25 °C. At the end of incubation, the amount of ABTS^·+^ was determined by absorbance at 415 nm (ε415nm = 36 mM^−1^.cm^−1^). The enzymatic reaction (0.2 mL) was evaluated in 50 mM Na-Acetate pH 5.0 and 50 mM NaCl, by adding the purified FHS-OGOX1 (4 nM) and OGs or the appropriate galacturonan oligomer (OG4, OG3) (15 µM) or, alternatively, by adding the purified FHS-CELLOX (16 nM) and the appropriate CD (CD4 or CD3) (15 µM). oxOG4 was prepared from OG4 as previously described^6^ whereas CD4 and CD3 were purchased from Megazyme (Bray, Ireland). For each galacturonan and CD stock solution (1 mM), the amount of reducing ends was confirmed by the reducing sugar-assay^28^ using different amounts of glucose as calibration curve. To evaluate the production of H_2_O_2_ in the ABTS^·+^-reduction assay, HRP was added to each reaction (1.25 µM). To determine the reduction of ABTS^·+^ over time, the absorbance at 415 nm from the enzymatic reactions was measured in continuous mode, subtracted to that of control reaction (basal mixture) and then converted into µmol ABTS^·+^ (ε415nm = 36 mM^−1^.cm^−1^). In parallel, the xylenol orange assay was used to measure the amount of H_2_O_2_ produced by FHS-OSOXs under the same reaction conditions [50 mM Na-Acetate pH 5.0 and 50 mM NaCl, pure FHS-OGOX1/CELLOX (4/16 nM) and the appropriate oligosaccharide (15 µM)]. All the analyses were performed in triplicates by using an Infinite^®^ M Nano200 spectrophotometer (Tecan AG, Männedorf, Switzerland).

### Evaluation of ABTS-oxidizing activity of fungal laccase in the presence of FHS-OSOXs and short oligosaccharides

The ABTS-oxidizing activity of laccase from *Trametes versicolor* (38429, Sigma-Aldrich, St. Louis, USA) was evaluated in 50 mM Na-Acetate pH 5.0 and 50 mM NaCl, by using the ABTS-oxidation assay (100 µM ABTS) in the presence of the four FHS-OGOX1/OG4, FHS-OGOX1/OGs, FHS-CELLOX/CD3 and FHS-CELLOX/CD4 combinations. The activity was assayed by using the laccase alone (5 µg.mL^−1^) or in the presence of FHS-OGOX1 (4 nM) and OGs/OG4 (15 µM), or in the presence of FHS-CELLOX (16 nM) and CD3/CD4 (15 µM) in a final reaction volume of 0.2 mL. For the ABTS-oxidation assay, the amount of ABTS^·+^ was spectrophotometrically measured at 25 °C following the absorbance at 415 nm, subtracted to that of control reaction (basal mixture) and converted into µmoles of ABTS^·+^ (ε415nm = 36 mM^−1^.cm^−1^). In parallel, the xylenol orange assay was used to measure the amount of H_2_O_2_ produced by FHS-OSOXs in the presence of laccase (5 µg.mL^−1^) under the same reaction conditions [100 µM ABTS, 50 mM Na-Acetate pH 5.0 and 50 mM NaCl, pure FHS-OGOX1/CELLOX (4/16 nM) and the appropriate oligosaccharide (15 µM)] in a final reaction volume of 0.1 mL. All the analysis were performed in triplicates at 25 °C.

### Analysis of residual CD3 by HPLC

The amount of residual CD3 upon oxidation with FHS-CELLOX was determined by HPLC. Due to the limit of detection of our HPLC analysis, the OSOX/oligosaccharide concentrations were increased by maintaining the same ratio of previously described reactions. In our experimental conditions, only CD3 was detectable whereas the corresponding (acidic) CELLOX-oxidized CD3 (i.e. Glc1–4βGlc1–4Gluconic acid^7^) was undetectable, likely retained by the column. For the analysis of residual CD3 in the presence of ABTS^·+^, FHS-CELLOX (0.7 µM) and CD3 (600 µM) were incubated without stirring for 0.5, 4, 8 and 16 min in a buffer composed of 50 mM Na-Acetate pH 5, 50 mM NaCl and 1 mM ABTS^·+^/ABTS (800/200 µM) (0.2 mL). For the analysis of residual CD3 in the control reaction (i.e. without ABTS^·+^), the enzymatic reactions were performed under the same reaction conditions but without the ABTS^·+^/ABTS mixture. In order to assess the effect of H_2_O_2_ on the activity of FHS-CELLOX, catalase from bovine liver (4 µg) (C1345, Sigma-Aldrich, St. Louis, USA) was added to the reaction. The reactions were performed at 25 °C and then heat-inactivated (90 °C × 10 min) before the analysis, except for the reaction with the shortest incubation time (i.e. 0.5 min) that was immediately injected. HPLC analysis was carried out using a Shimadzu LC-2030 Plus Prominence-i (Japan) system equipped with a Shimadzu Differential Refractive Index Detector (RID-20A). Chromatographic separation was carried out by using a Knauer Eurokat-Pb column (300 × 4 mm, 10 µm particle size). The mobile phase consisted of isocratic elution using distilled water (eluent A). The injection volume for all samples was 40 µL, whereas the flow rate and chromatographic separation time were 0.4 mL.min^−1^ and 15 min, respectively. Eluent A was filtered through 0.2 μm pore size filter. The column temperature was maintained at 75 °C. Shimadzu LabSolutions software was used for data acquisition, instrument control, and data analysis. The chromatograms shown in Fig. 5 were obtained by subtracting the chromatograms of complete reaction to that of basal reaction (i.e. reaction without the substrate).

## Supplementary Information

Supplementary Figures.

## Data Availability

All relevant data are included in the article and/or its Supplementary Material. The datasets used and/or analyzed during the current study are available from M.B. on reasonable request.
