# Supplementary material for: Radical cation scavenging activity of berberine bridge enzyme-like oligosaccharide oxidases acting on short cell wall fragments
Source: Sci Rep. 2023 Mar 13;13:4123. doi: 10.1038/s41598-023-31335-y (PMC10011498; doi:10.1038/s41598-023-31335-y)
Supplement: Supplementary file 1 — Supplementary Figures. [file 41598_2023_31335_MOESM1_ESM.pdf]

SUPPLEMENTARY MATERIAL

RADICAL CATION SCAVENGING ACTIVITY OF BERBERINE BRIDGE ENZYME-LIKE OLIGOSACCHARIDE OXIDASES ACTING ON SHORT CELL WALL FRAGMENTS.

Anna Scortica[1,§], Valentina Scafati[1,§], Moira Giovannoni[1,§], Manuel Benedetti[1,*], Benedetta Mattei[1]

[1]Department of Life, Health and Environmental Sciences, University of L'Aquila, 67100 L'Aquila, Italy

*correspondence: manuel.benedetti@univaq.it, Department of Life, Health and Environmental Sciences, University of L'Aquila, 67100 L'Aquila, Italy; phone +39 0862433272.

[§], These authors equally contributed to this work.

TABLE OF CONTENTS

Supplementary Figures (Fig. S1-S4)

Figure S1. Heterologous expression of FHS-OGOX1 in *P. pastoris*.

Figure S2. Production of ABTS requires the activity of FHS-OGOX1 on OG4.

Figure S3. Production of ABTS and $ABTS^{\bullet +}$ by the activity of FHS-OGOX1 on different OG-oligomers in the presence of HRP.

Figure S4. Production of H_2O_2 by the activity of different OSOX/oligomer combinations in the presence of laccase.

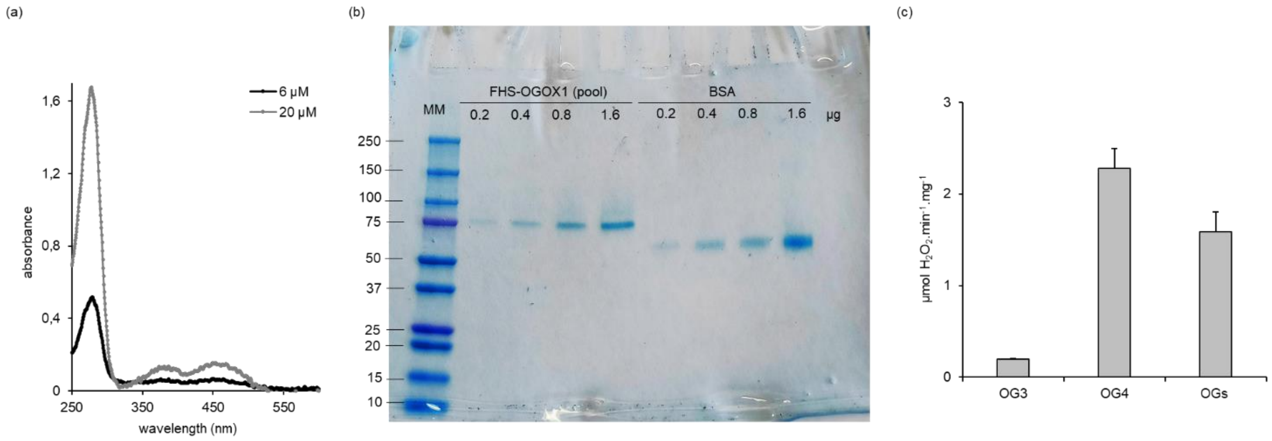

Figure S1. Heterologous expression of FHS-OGOX1 in *P. pastoris*. (a) UV-visible spectrum of pure FHS-OGOX1 at two different concentrations (6 µM and 20 µM) as determined by UV-absorbance. (b) SDS-PAGE/Coomassie blue staining analysis of different amounts of pure FHS-OGOX1 as determined by UV-absorbance. Same amounts of BSA were used as control. Molecular weight marker (MM) is also reported. (c) Oxidizing activity of FHS-OGOX1 (µmol $H_2O_2.min^{-1}.mg^{-1}$) at pH 5.0 using OG-oligomers with different length as substrates. Values are mean ± SD (N=3). [FHS-OGOX1: flag-his-sumoylated oligogalacturonide-oxidase 1, OGs: oligogalacturonides, OG4: tetra-galacturonic acid, OG3: tri-galacturonic acid].

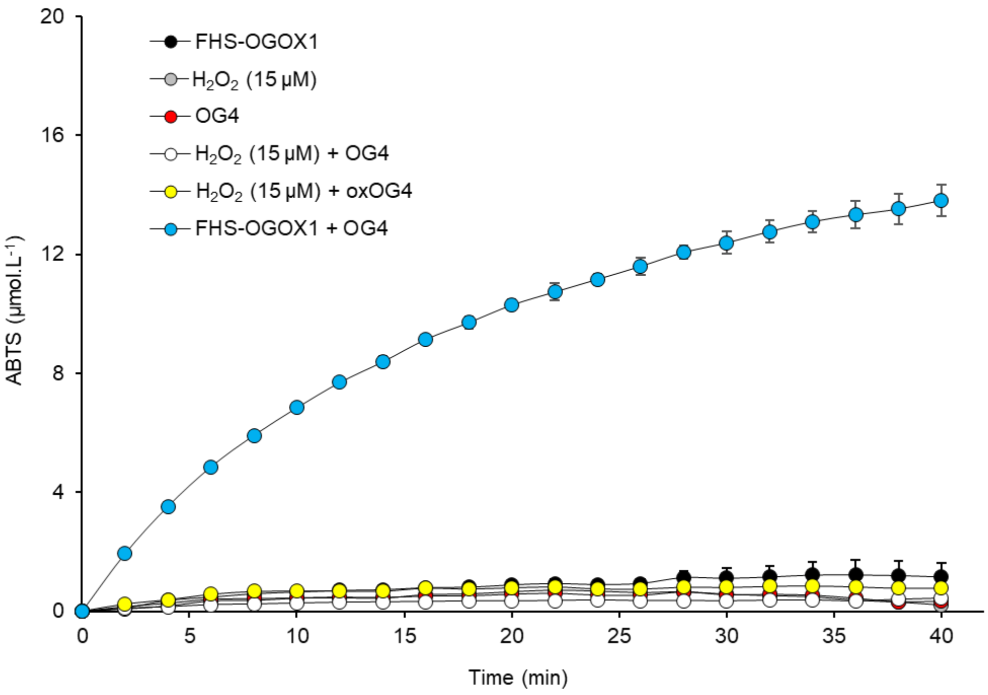

Figure S2. Production of ABTS requires the activity of FHS-OGOX1 on OG4. Production of ABTS (µmol.L^{-1}) over time at pH 5.0 using different combinations of substrates and reactants. FHS-OGOX1 and OG4 were used at 4 nM and 15 µM, respectively. Values are mean ± SD (N=3). [ABTS: 2,2'-azino-bis (3-ethylbenzothiazoline-6-sulfonic acid), FHS-OGOX1: flag-his-sumoylated oligogalacturonide-oxidase 1, OG4: tetra-galacturonic acid, oxOG4: oxidized tetra-galacturonic acid].

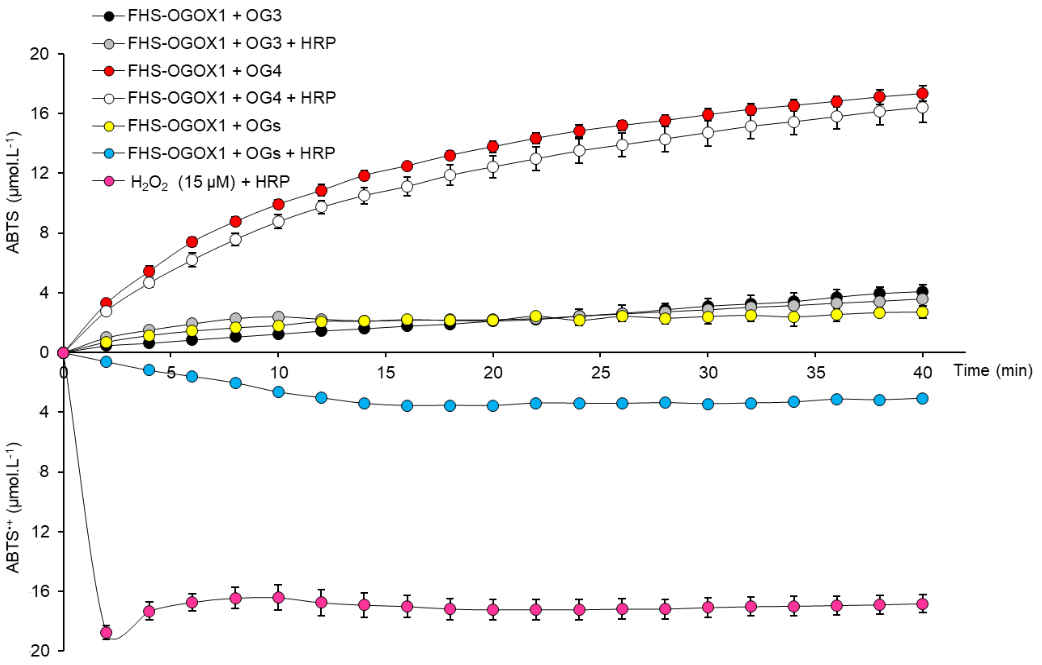

Figure S3. Production of ABTS and ABTS$^{\bullet+}$ by the activity of FHS-OGOX1 on different OG-oligomers in the presence of HRP. Production of ABTS and ABTS$^{\bullet+}$ (µmol.L^{-1}) over time at pH 5.0 by the activity of FHS-OGOX1 (4 nM) and OG3 (15 µM), OG4 (15 µM) and OGs (15 µM) in the presence of HRP (+ HRP, 0.05 g.L^{-1}). [H_2O_2 + HRP] is reported as positive control of HRP-mediated oxidation of ABTS. Values are mean ± SD (N=3). [ABTS: 2,2'-azino-bis (3-ethylbenzothiazoline-6-sulfonic acid), FHS-OGOX1: flag-his-sumoylated oligogalacturonide-oxidase 1, HRP: horseradish peroxidase VI-type, OGs: oligogalacturonides, OG4: tetra-galacturonic acid, OG3: tri-galacturonic acid].

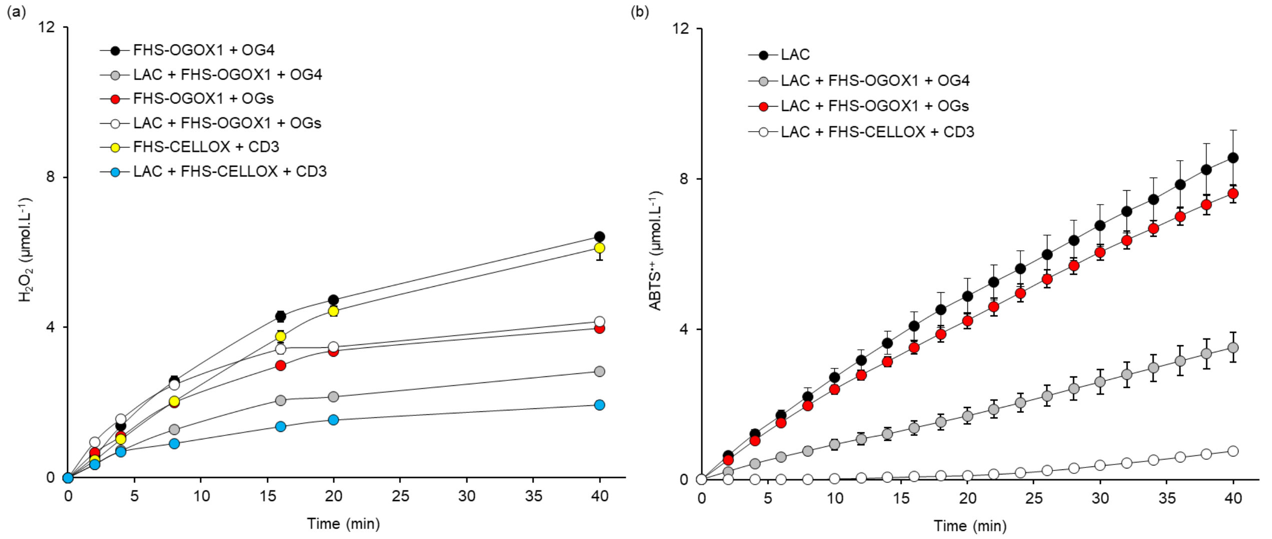

Figure S4. Production of H_2O_2 by the activity of different OSOX/oligomer combinations in the presence of laccase. (a) Production of H_2O_2 (µmol.L^{-1}) over time at pH 5.0 by the activity of FHS-OGOX1/OG4, FHS-OGOX1/OGs and FHS-CELLOX/CD3 combinations in the presence of laccase (5 µg.mL^{-1}) as determined by the xylenol orange assay. (b) Production of ABTS$^{\bullet+}$ over time by laccase alone and in the presence of the same OSOX/oligomer combinations shown in (a) (extrapolated from Fig. 4). FHS-OGOX1, FHS-CELLOX and each oligomer were used at 4 nM, 16 nM and 15 µM, respectively. Values are mean ± SD (N=3). [ABTS: 2,2'-azino-bis (3-ethylbenzothiazoline-6-sulfonic acid), CD3: cellotriose, FHS-CELLOX: flag-his-sumoylated cellodextrin-oxidase, FHS-OGOX1: flag-his-sumoylated oligogalacturonide-oxidase 1, LAC: laccase from *T. versicolor*, OGs: oligogalacturonides, OG4: tetra-galacturonic acid].
